# Impact of an Educational Intervention on Inhalation Technique and Self-Reported Medication Adherence in Adults With Asthma and COPD in a Primary Care Unit

**DOI:** 10.7759/cureus.117426

**Published:** 2026-10-04

**Authors:** Ana Rodrigues, Susana Martins, Ana Cabral, Isabel Vitória Figueiredo, Margarida Castel-Branco

**Affiliations:** 1 Laboratory of Pharmacology and Pharmaceutical Care, Faculty of Pharmacy, University of Coimbra, Coimbra, PRT; 2 Family Health Unit A Ribeirinha, Local Health Unit of Guarda, Guarda, PRT

**Keywords:** asthma, chronic obstructive pulmonary disease, inhalation technique, inhaler devices, therapy adherence

## Abstract

Introduction: Chronic obstructive pulmonary disease (COPD) and asthma are chronic respiratory diseases characterized by airflow restriction and heterogeneous clinical expressions. Inhaled therapy is the cornerstone of management, delivering medication directly to the respiratory tract using inhaler devices, such as pMDIs (pressurized metered dose inhalers), sDPIs (single-dose dry powder inhalers), mDPIs (multiple-dose DPIs), and SMIs (soft mist inhalers). Treatment effectiveness relies on a correct inhalation technique to guarantee optimal drug delivery and adequate treatment adherence to sustain disease control. In this context, prospective primary care data remain scarce regarding how face-to-face educational instruction influences inhaler handling and multidimensional adherence behaviors.

Objective: The primary objective was to compare pre- and post-intervention inhalation technique in patients with COPD or asthma attending the primary healthcare unit. The secondary objective was to evaluate treatment adherence through a self-reported questionnaire.

Methods: This was a prospective interventional study, approved by the Institutional Ethics Committee and conducted at a Portuguese primary healthcare unit from April to August 2024. Adult patients with COPD or asthma using at least one previously prescribed inhaler were included. The intervention consisted of assessing inhalation technique using device-specific checklists before and two months after face-to-face instruction on the correct technique. Each 30- to 45-minute educational session included 15 minutes dedicated exclusively to demonstrating and correcting the inhalation technique. Self-reported therapy adherence was evaluated through the Medication Adherence Universal Questionnaire (MAUQ) before and after the intervention. Following data insertion into a Microsoft Excel (Microsoft Corp., Redmond, WA, USA) database, statistical analysis was performed.

Results: From a total of 76 patients (29 COPD, 47 asthma) initially assessed across 99 inhaler devices, 23 were lost to follow-up (5 COPD, 18 asthma). Ultimately, 53 patients completed the study, corresponding to 69 evaluated inhaler devices. Regarding inhalation technique, SMIs showed the highest proportion of inhalation technique errors (51.11%), followed by mDPIs (37.50%) and pMDIs (33.20%). Following the educational intervention, a significant improvement was noted in common errors, specifically "Failure to exhale forcefully prior to actuation" and "Not holding breath for at least 5 seconds," among asthma patients. The number of errors decreased in 14 (50.00%) COPD and 27 (71.05%) asthma techniques, reaching statistical significance only within the asthma group (p<0.001, Wilcoxon signed-rank test). Error proportion decreased significantly in mDPIs (p<0.001, Wilcoxon signed-rank test). In terms of treatment adherence, although MAUQ total scores revealed no statistical variations within groups before and after the intervention (COPD p=0.125, asthma p=0.195, Wilcoxon signed-rank test), the "Lack of Discipline" dimension improved significantly in both groups (COPD p=0.041, asthma p=0.006, Wilcoxon signed-rank test).

Conclusion: Structured teaching and regular review of inhalation techniques in primary care allow healthcare professionals to identify and correct device handling errors that emerge over time. Nevertheless, a more comprehensive, multifaceted intervention is required to effectively promote long-term treatment adherence.

## Introduction

Chronic obstructive pulmonary disease

Chronic obstructive pulmonary disease (COPD) is the fourth leading cause of death globally, according to data from the World Health Organization [1]. It is defined as a chronic respiratory illness characterized by persistent airflow obstruction, which leads to symptoms such as cough, sputum production, and dyspnea [2]. While tobacco smoking remains the primary risk factor in high-income countries, indoor air pollution, occupational exposure, and respiratory infections contribute to more than 60% of COPD cases in lower-middle-income countries [1]. Although COPD already places a substantial global disease burden, epidemiological projections estimate that the number of individuals diagnosed with COPD in high-income countries will reach 166 million by 2050 [1,3]. In Portugal, COPD is the eighth leading cause of death, with a documented prevalence of 14.2% in adults aged over 40 years [4].

Asthma

Asthma is a long-term chronic inflammatory disease affecting both children and adults. It results from complex interactions between environmental, genetic, and individual factors. Asthma symptoms, including coughing, dyspnea, chest tightness, and wheezing, occur intermittently and vary in intensity [5]. It remains a major noncommunicable disease worldwide, affecting an estimated 363 million people in 2023. Urbanization is associated with an increased prevalence of asthma, possibly due to multiple lifestyle factors [6]. Furthermore, a recent nationwide, population-based survey assessing asthma prevalence in Portugal reported that the condition affects 7.1% of Portuguese adults [7].

In Portuguese primary healthcare, managing obstructive airway diseases remains a major clinical challenge due to underdiagnosis and suboptimal treatment adherence. The scarcity of interventional research in primary care settings emphasizes the need for prospective studies to assess the impact of structured patient education, specifically focusing on inhaler technique instruction and long-term adherence.

Inhaled therapy, inhaler devices, and inhalation technique

The management of COPD and asthma relies on inhaled therapy, minimizing the risk of adverse effects when compared with oral administration. Optimizing treatment outcomes depends on two related yet distinct components: inhalation technique, which involves the correct use of inhaler devices to ensure proper pulmonary drug deposition, and treatment adherence, which reflects the patient's behavioral compliance with the prescribed treatment [8,9]. A wide range of inhaler devices is currently available on the market, differing primarily in their delivery systems: pressurized metered dose inhalers (pMDIs), single-dose dry powder inhalers (sDPIs), multiple-dose DPIs (mDPIs), and soft mist inhalers (SMIs). Correctly handling these devices requires common steps (forceful exhalation prior to actuation, vigorous inhalation for DPIs or slow inhalation for pMDIs, and holding breath for at least 5 seconds) as well as device-specific steps [9,10]. Consequently, errors occurring in common steps can be replicated among devices, thereby compromising treatment effectiveness. 

Therapy adherence

Therapy adherence is a complex, multidimensional concept that determines therapeutic success [11]. Previous studies have reported that 30% to 70% of adults with asthma are non-adherent. Similarly, medication non-adherence remains a critical issue in COPD, with estimated rates varying between 22% and 93% [12,13].

The international guidelines provided by the Global Initiative for Chronic Obstructive Lung Disease (GOLD) and Global Initiative for Asthma (GINA) recommend regular reassessment of inhalation technique and treatment adherence to identify the causes of inadequate clinical response [14,15]. Consequently, the primary objective of this study was to compare inhalation technique and identify device-specific errors in patients with COPD or asthma before and after an educational intervention in a primary healthcare unit. The secondary objective was to evaluate treatment adherence using a self-reported questionnaire designed for chronic diseases.

This study was previously presented as a short lecture presentation entitled "Assessment of Inhalation Technique and Therapy Adherence in a Primary Care Unit" at the 25th WONCA World Conference, Lisbon, September 17-21, 2025.

## Materials and methods

This prospective, single-arm, pre-post interventional study was conducted between April and August 2024 in a primary healthcare setting located in Guarda, Portugal, among patients diagnosed with COPD and asthma. The study protocol was approved by the Ethics Committee of the Local Health Unit of Guarda (approval number: UI_A50_2023-103/2023, dated January 30, 2024).

The list of individuals coded as R95 (COPD) and R96 (asthma) according to the International Classification of Primary Care-2 was extracted from the institutional electronic health record database used in the healthcare unit. The database was accessed by a general practitioner collaborating in the study. In addition to names and telephone numbers, the retrieved list included the medical record number, national health service number, age, sex, and neighborhood.

All patients aged 18 years or older with a confirmed diagnosis of COPD or asthma were invited via telephone and recruited after an initial semi-structured interview carried out to determine their eligibility. Patients using at least one inhaler device were eligible for inclusion, whereas first-time users (defined as patients who had initiated their prescribed inhaler less than one month prior to study enrollment) were excluded to ensure that baseline assessment reflected established real-world, long-term handling habits. The presence of cognitive impairment constituted an exclusion criterion. During the initial semi-structured interview, patients were screened for self-perceived or clinically diagnosed conditions affecting memory or learning.

The study comprised a pre- and post-intervention phase, each consisting of a single 30- to 45-minute session per patient, receiving the exact same face-to-face educational intervention. Within each session, 15 minutes were dedicated exclusively to evaluating and demonstrating the inhalation technique. Initially, patients demonstrated their device handling while the healthcare professional assessed their performance using specific checklists. Each step was evaluated dichotomously as "yes" (correctly executed) or "no" (incorrectly executed or omitted), and the sum of failed steps was used to quantify handling errors per device. Following a standardized procedure, the correct inhalation technique (intervention) was demonstrated by the healthcare professional alongside an explanation of the device-specific mechanism and recommendations regarding the maintenance and proper storage of inhaler devices. Patients were then asked to replicate the inhalation technique taught, returning two months later for a reassessment. The two-month interval was chosen due to the documented decay of inhalation technique skills over one to three months following educational interventions. Therapy adherence was evaluated through the Medication Adherence Universal Questionnaire (MAUQ), as seen in Figure 1, which was applied before and after the intervention. The questionnaire was validated and applied in Portuguese. To ensure consistency and full understanding, the MAUQ was administered by the healthcare professional through a structured face-to-face reading, while participants visually followed along with a copy of the questionnaire. All the responses were recorded directly by the healthcare professional.

**Figure 1:**
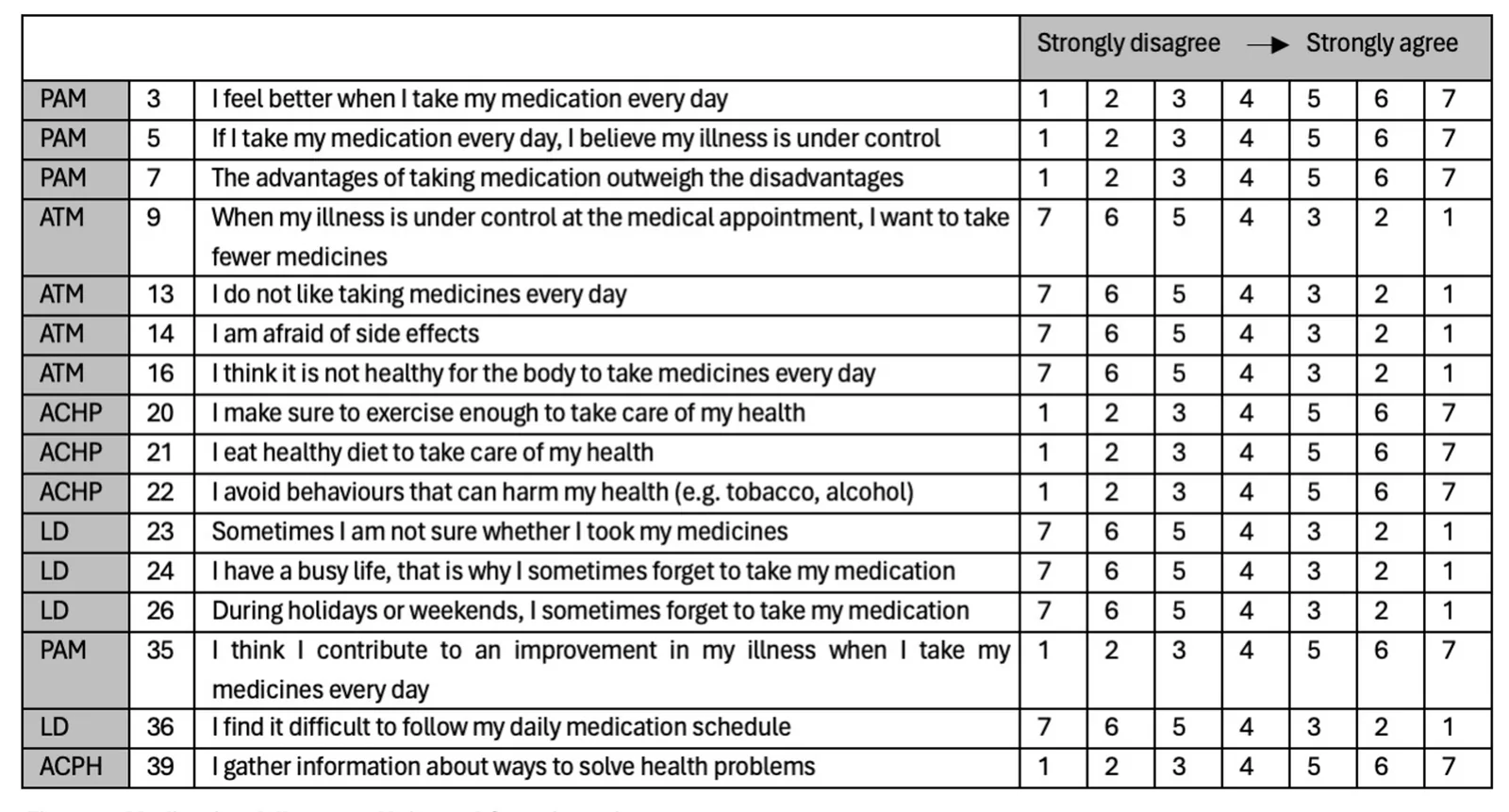
Medication Adherence Universal Questionnaire. PAM: positive attitudes towards healthcare and medication, LD: lack of discipline, ATM: aversion towards medication, ACHP: active coping with health problems. *Source*: Reproduced with permission from Cabral et al. [16].

The MAUQ was designed to evaluate four distinct dimensions: positive attitudes towards healthcare and medication (PAM), lack of discipline (LD), aversion towards medication (ATM), and active coping with health problems (ACHP), with each dimension comprising four items. For each item, responses are scored on a seven-point Likert scale ranging from "Totally disagree" to "Totally agree." A maximum score of 28 points can be achieved per dimension, yielding a cumulative maximum questionnaire score of 112 points. A reverse-scored seven-point Likert scale is used for items in the LD and ATM dimensions. Consequently, lower scores in these dimensions indicate the presence of barriers to adherence, while higher scores in the PAM and ACHP dimensions reflect behaviors and attitudes that favor adherence.

Data collection

After obtaining informed written consent, clinical data (diagnosis, years of diagnosis, prescribed inhaler, and inhaler classification) and demographic characteristics (age and sex) were collected. All inhalation techniques were evaluated by the same healthcare professional using an adapted version of the checklists developed by the Portuguese Society of Allergology and Clinical Immunology. These checklists are aligned with international guidelines as well as manufacturers' instructions, incorporating device-specific critical steps to identify handling errors, such as dose preparation and inhalation flow dynamics. A total of 11 distinct checklists were used, specifically tailored for pMDIs without a valved holding chamber (VHC), pMDIs with a VHC in single-breath technique, pMDIs with a VHC in multiple-breath technique, sDPIs (Breezhaler® (Novartis Europharm Limited, Dublin, Ireland), Handihaler® (Boehringer Ingelheim, Ingelheim, Germany), Rotacaps® (Cipla Ltd., Mumbai, India), and Aerolizer® (Novartis Europharm Limited, Dublin, Ireland)), mDPIs (Diskus® (GlaxoSmithKline plc, London, UK), Ellipta® (GlaxoSmithKline plc, London, UK), Turbohaler® (AstraZeneca PLC, Cambridge, UK), Novolizer® (Viatris Inc., Canonsburg, PA, USA)/Genuair® (AstraZeneca PLC, Cambridge, UK), Spiromax® (Teva Pharmaceutical Industries Ltd., Tel Aviv, Israel), and Easyhaler® (Orion Corporation, Espoo, Finland)), and SMIs (Respimat® (Boehringer Ingelheim)). Checklists were variable, containing seven to 14 steps, depending on the device, which were required to demonstrate a correct inhalation technique.

Statistical data analysis

All collected data were entered into a Microsoft Excel (Microsoft Corp., Redmond, WA, USA) database and analyzed using IBM SPSS Statistics for Windows, Version 29.0 (Released 2022; IBM Corp., Armonk, NY, USA). Qualitative variables, including gender and inhaler classification, were expressed as frequencies and percentages (%), while quantitative variables, including age, years of diagnosis, and MAUQ score, were expressed as median and interquartile range (IQR).

To assess the effect of the intervention within patients' groups, pre- and post-intervention evaluations were compared regarding the overall MAUQ score, individual MAUQ dimension scores, and the proportion of handling errors of inhalation techniques. Data normality was assessed using the Shapiro-Wilk test. To compare continuous outcome variables within patients’ groups, the non-parametric Wilcoxon signed-rank test for paired samples was used for non-normally distributed data. The unit of analysis for inhalation technique evaluation was each specific inhaler device per patient, pairing baseline and follow-up observations. Techniques from patients lost to follow-up or lacking post-intervention assessments were excluded from the paired analysis.

## Results

A total of 115 patients diagnosed with COPD and 364 with asthma were initially identified at the primary healthcare unit. Of these, 126 patients were excluded for not fulfilling the inclusion criteria, and 66 were excluded for other reasons. Of the 287 eligible patients, 97 refused to participate, and other 97 did not answer the phone calls. Although 93 individuals initially agreed to enter the study, only 76 attended the baseline assessment, as demonstrated in Figure 2.

**Figure 2:**
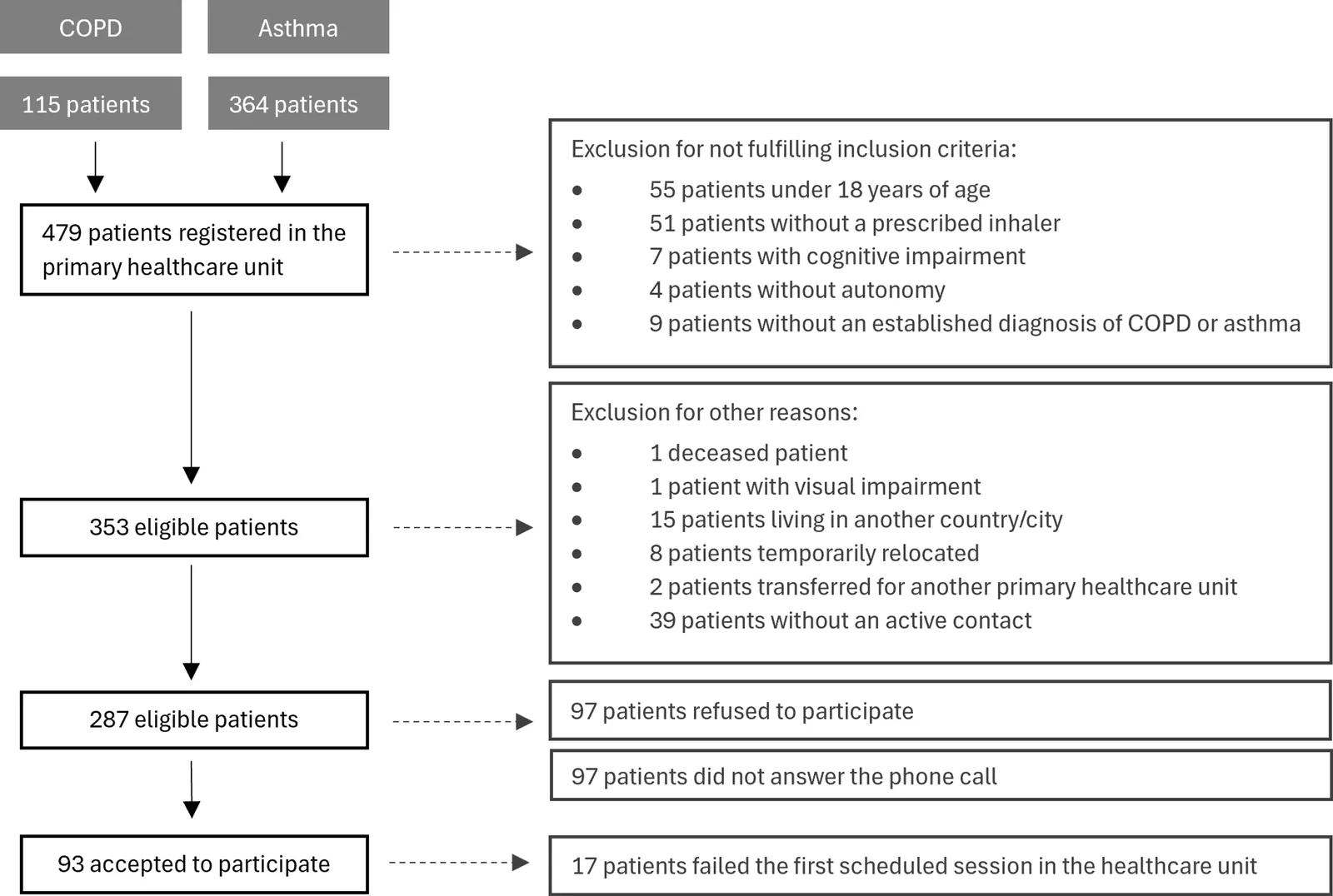
Flowchart of the study population. COPD: chronic obstructive pulmonary disease.

Demographic and clinical parameters

Table 1 outlines the demographic and clinical parameters of the study population. A total of 76 patients were enrolled in the study, of whom 29 were diagnosed with COPD. Among COPD patients, the median age was 70 years (Q1-Q3: 61-75.5), with male participants representing more than half of the group (75.86%). The median age in asthma group was 49 years (Q1-Q3: 37-64), and the majority of the 47 patients were females (65.96%). In both groups, the majority of the participants had been diagnosed at least five years ago and had a single prescribed inhaler (Table 1). Following the intervention, 53 patients (24 COPD and 29 asthma) completed the follow-up period.

**Table 1:** Demographic and clinical characteristics of study patients. Q1: first quartile; Q3: third quartile.

	COPD group (n= 29)	Asthma group (n=47)
Age, years (median, Q1-Q3)	70, Q1-Q3: 61-75.5	49, Q1-Q3: 37-64
Gender, n (%)		
Female	7 (24.14)	31 (65.96)
Male	22 (75.86)	16 (34.04)
Years of diagnosis, n (%)		
<1 year	1 (3.44)	0 (0.00)
1–5 years	10 (34.48)	5 (10.63)
5–10 years	9 (31.03)	1 (2.13)
>10 years	9 (31.03)	41 (87.23)
Number of prescribed inhalers, n (%)		
1	23 (79.31)	35 (74.47)
2	5 (17.24)	8 (17.02)
3	1 (3.44)	4 (8.51)

A total of 99 inhaler devices (36 COPD, 63 asthma) were evaluated. As demonstrated in Figure 3, DPIs represented the most frequently used device in both groups, with 24 devices (66.66%) in the COPD group and 39 devices (61.90%) in the asthma group. The preferred delivery systems varied between the two groups. Among patients using DPIs, Breezhaler® was the most frequently used device in the COPD group, with 11 devices (45.83%), whereas Turbohaler® was the most frequently used device in the asthma group, with 20 devices (51.28%).

**Figure 3:**
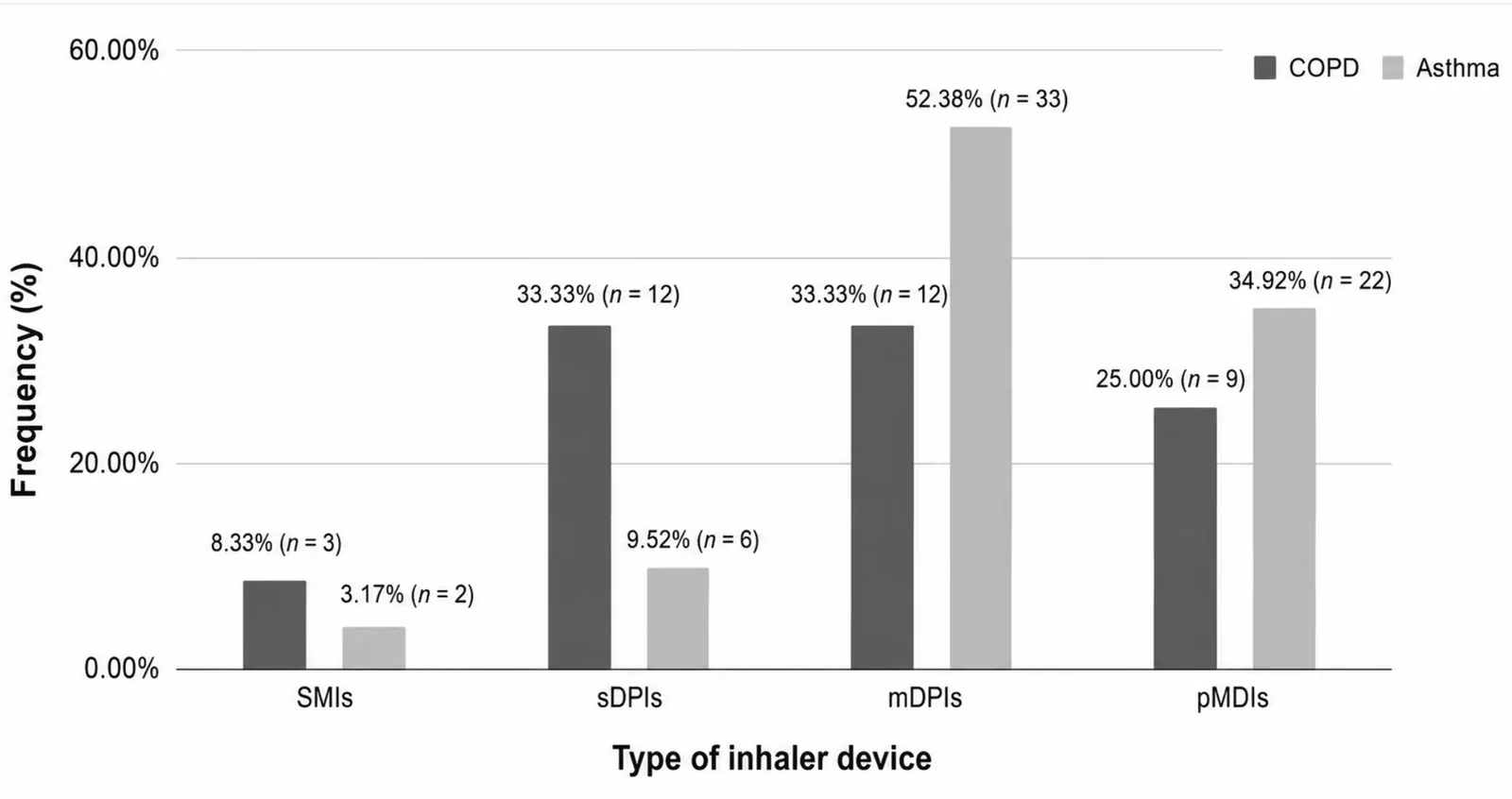
Frequency of each type of inhaler device presented in the study, according to diagnosis. SMIs: soft mist inhalers; sDPIs: single-dose dry powder inhalers; mDPIs: multiple-dose dry powder inhalers; pMDIs: pressurized metered dose inhalers; COPD: chronic obstructive pulmonary disease.

Pre-intervention versus post-intervention

Figure 4 presents the flowchart of the study. Of the 99 inhalation techniques (36 from the 29 patients with COPD and 63 from the 47 patients with asthma) initially assessed, only 66 were re-evaluated at the post-intervention phase. A total of 23 patients were lost to follow-up at the two-month assessment. Among the re-evaluated techniques, three were excluded due to changes in the prescribed device, which led to a different inhaler and consequently a different inhalation technique being evaluated after the intervention. In COPD, 28 inhalation techniques were reassessed among the 24 remaining patients, while in asthma 38 techniques were reassessed among 29 patients remaining in the study. Regarding the medication adherence assessment, a total of 76 MAUQ questionnaires were initially applied. However, three were excluded due to nonconformity (26 COPD, 47 asthma). After the intervention, 53 questionnaires were reapplied (24 COPD, 29 asthma). The questionnaires from the three patients who experienced changes in their prescribed inhaler were included in the adherence analysis alongside the rest of the group.

**Figure 4:**
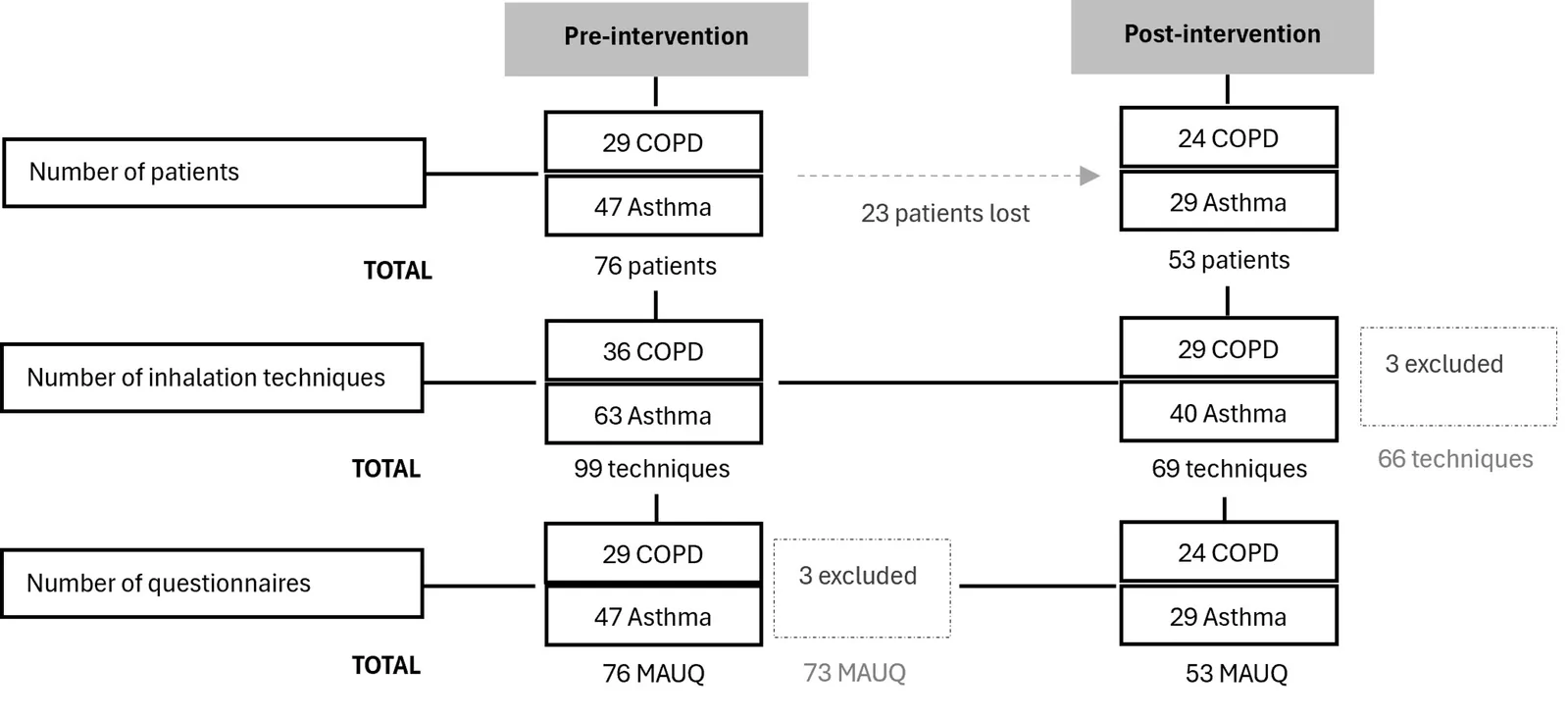
Flowchart of evaluated patients, techniques, and questionnaires before and after the intervention. COPD: chronic obstructive pulmonary disease; MAUQ: Medication Adherence Universal Questionnaire.

Assessment of inhalation technique

At baseline, "Failure to exhale forcefully prior to actuation" was the most frequently observed common error, occurring in 19 (52.78%) of COPD inhalation techniques, while "Failure to inhale vigorously for DPIs or slow for pMDIs" was the most prevalent error detected in 29 (46.03%) of the asthma inhalation techniques, as shown in Table 2. Afterwards, "Failure to exhale forcefully prior to actuation" and "Failure to inhale vigorously for DPIs or slow for pMDIs" remained the most frequent errors within the COPD and asthma groups, occurring in 11 (39.29%) and 7 (18.42%), respectively.

**Table 2:** Frequency of common pre- and post-intervention errors on inhalation technique of pMDIs, sDPIs, mDPIs, and SMIs, according to diagnosis. pMDIs: pressurized metered dose inhalers, sDPIs: single-dose dry powder inhalers, mDPIs: multiple-dose dry powder inhalers, SMIs: soft mist inhalers.

Errors identified in common steps	Pre-intervention: COPD, n (%)	Pre-intervention: Asthma, n (%)	Post-intervention: COPD, n (%)	Post-intervention: Asthma, n (%)
pMDIs, sDPIs, mDPIs, SMIs	36 (100.00%)	63 (100.00%)	28 (100.00%)	38 (100.00%)
Failure to exhale forcefully prior to actuation	19 (52.78%)	20 (31.75%)	11 (39.29%)	1 (2.63%)
Failure to inhale vigorously for DPIs or slow for pMDIs	5 (13.89%)	29 (46.03%)	4 (14.29%)	7 (18.42%)
Not holding breath for at least 5 seconds	14 (38.89%)	12 (19.05%)	3 (10.71%)	2 (5.26%)

Comparison between pre- and post-intervention errors observed across the common steps showed a statistically significant improvement in asthmatic patients. The frequency of errors related to “Failure to exhale forcefully prior to actuation” (p<0.001, Wilcoxon signed-rank test) and “Not holding breath for at least 5 seconds” (p=0.014, Wilcoxon signed-rank test) decreased from 20 (31.75%) to 1 (2.63%) and from 12 (19.05%) to 2 (5.26%), respectively. In the COPD group, errors related to "Not holding breath for at least 5 seconds" decreased from 14 (38.89%) to 3 (10.71%) (p=0.02, Wilcoxon signed-rank test).

Across the various types of devices evaluated, errors were also observed during device-specific steps. Although a wide range of devices was analyzed, this section highlights the most clinically relevant findings, while detailed data regarding the specific errors observed are summarized in Table 3. To avoid overinterpretation in small device subgroups, findings are presented primarily as absolute frequencies (n).

**Table 3:** Frequency of specific pre- and post-intervention errors on inhalation technique of pMDIs, sDPIs, mDPIs, and SMIs, according to diagnosis. Percentages were omitted for specific error steps to avoid misinterpretation resulting from small subgroup denominators. pMDIs: pressurized metered dose inhalers; VHC: valve holding chamber; sDPIs: single-dose dry powder inhalers; mDPIs: multiple-dose dry powder inhalers; SMIs: soft mist inhalers.

Errors identified in specific steps	Pre-intervention: COPD, n (%)	Pre-intervention: Asthma, n (%)	Post-intervention: COPD, n (%)	Post-intervention: Asthma, n (%)
pMDIs	5	21	3	9
Failure to remove the cap prior to device use	0	11	2	4
pMDIs-VHC	4	1	2	1
Failure to connect the inhaler to the VHC	3	0	1	0
Failure to place the VHC mouthpiece in the mouth or properly fit the mask around the nose and mouth	3	0	1	0
sDPIs	12	6	10	3
Failure to extract the capsule from its packaging and insert it into the capsule chamber	0	1	-	-
Failure to pierce the capsule before inhalation	2	0	1	0
Failure to release the piercing buttons before inhalation	2	0	1	0
Failure to remove the capsule and check for remaining powder in the capsule chamber	2	0	1	0
mDPIs (Diskus®)	-	3	-	3
Failure to load the dose by incorrectly sliding the lever	-	1	-	2
Not sliding the outer cover in the opposite direction as far as possible until it clicks into place to close the device	-	-	-	1
mDPIs (Ellipta®)	6	6	5	4
mDPIs (Turbohaler®)	1	20	1	13
Failure to remove the protective cap before using the inhaler	0	1	-	-
Failure to hold the inhaler upright with the twisting grip at the bottom	0	5	0	1
Failure to twist the grip as far as it will go in one direction and then back in the opposite direction until a click is heard	0	4	0	1
mDPIs (Genuair®/Novolizer®)	3	1	3	1
mDPIs (Spiromax®)	1	2	1	1
Failure to hold the inhaler with the protective mouthpiece cap at the bottom	1	0	-	-
Failure to open the protective mouthpiece cap until a click is heard	1	0	-	-
mDPIs (Easyhaler®)	1	1	1	1
SMIs	3	2	2	2
Failure to position the inhaler upright while keeping the mouthpiece cover closed	3	2	1	1
Failure to twist the grip in the indicated direction while holding the inhaler upright	2	2	1	1
Failure to open the protective cover immediately before inhalation	2	1	1	1

In pMDIs, errors were predominantly observed among patients with asthma, reflecting the widespread use of this type of device in this group. Among the 19 inhalation maneuvers performed by asthmatic patients where agitation was essential, “Not shaking the device before inhalation” was detected in 16 cases, at baseline. Following the intervention, the error was detected in five out of the eight remaining inhalation techniques. In contrast, patients diagnosed with COPD using a pMDI were evaluated based on checklists requiring attachment to a VHC. At baseline, three out of four COPD inhalation techniques with pMDI - VHC failed the steps related to connecting and properly positioning the VHC. Following the intervention, two patients remained in this group, of whom one continued to display this error.

Regarding the evaluated sDPIs, which were mainly used by the COPD group, several handling steps associated with the inhalation technique exhibited frequent errors. Specifically, in two of 12 inhalation techniques, failures were observed in steps related to piercing and removing the inhalation capsule at baseline. At the two-month follow-up assessment, the prevalence of these errors persisted in one of the 10 evaluated inhalation techniques.

Multiple-dose DPIs exhibit variations regarding their powder reservoirs. Consequently, different inhalers were evaluated using device-specific checklists within the mDPIs class. Errors detected in Turbohaler® inhalation techniques occurred mainly among asthma group and during the initial three steps involving dose preparation. Handling difficulties persisted in one of the remaining 13 inhalation techniques at follow-up.

The baseline assessment of SMIs exhibited failures in the three initial steps related to dose preparation. During the post-intervention phase, handling errors identified in the single affected COPD maneuver were successfully eliminated. Similarly, within the asthma group, an improvement in the dose preparation steps was noted in one of the evaluated inhalation techniques.

Overall, a consistent decline in the frequency of errors was detected across the inhalation maneuvers performed by patients diagnosed with COPD. In 27 inhalation techniques performed by patients with asthma, the error rate decreased, achieving statistical significance (p<0.001, Wilcoxon signed-rank test).

Error proportion with inhaler devices

To account for differences in device complexity and sample sizes across inhaler devices, error rates were standardized by calculating an overall error proportion for each device before and after the intervention using the following formula:

Error proportion (%) = [sum of the number of errors committed per demonstration/(number of checklist steps × total number of demonstrations per device)] × 100

Statistical comparisons through Wilcoxon signed-rank test were subsequently applied to these standardized error proportions.

Figure 5 illustrates that the highest proportion of errors at baseline was observed with SMIs (51.11%), followed by Diskus® and Spiromax® (37.50%) and pMDIs (33.20%), whose calculation was based on the sum of errors recorded across the three checklists used to evaluate pMDIs, with and without a spacer. In the post-intervention phase, as demonstrated in Figure 6, SMIs remained the inhaler devices with highest frequency of errors (30.55%).

**Figure 5:**
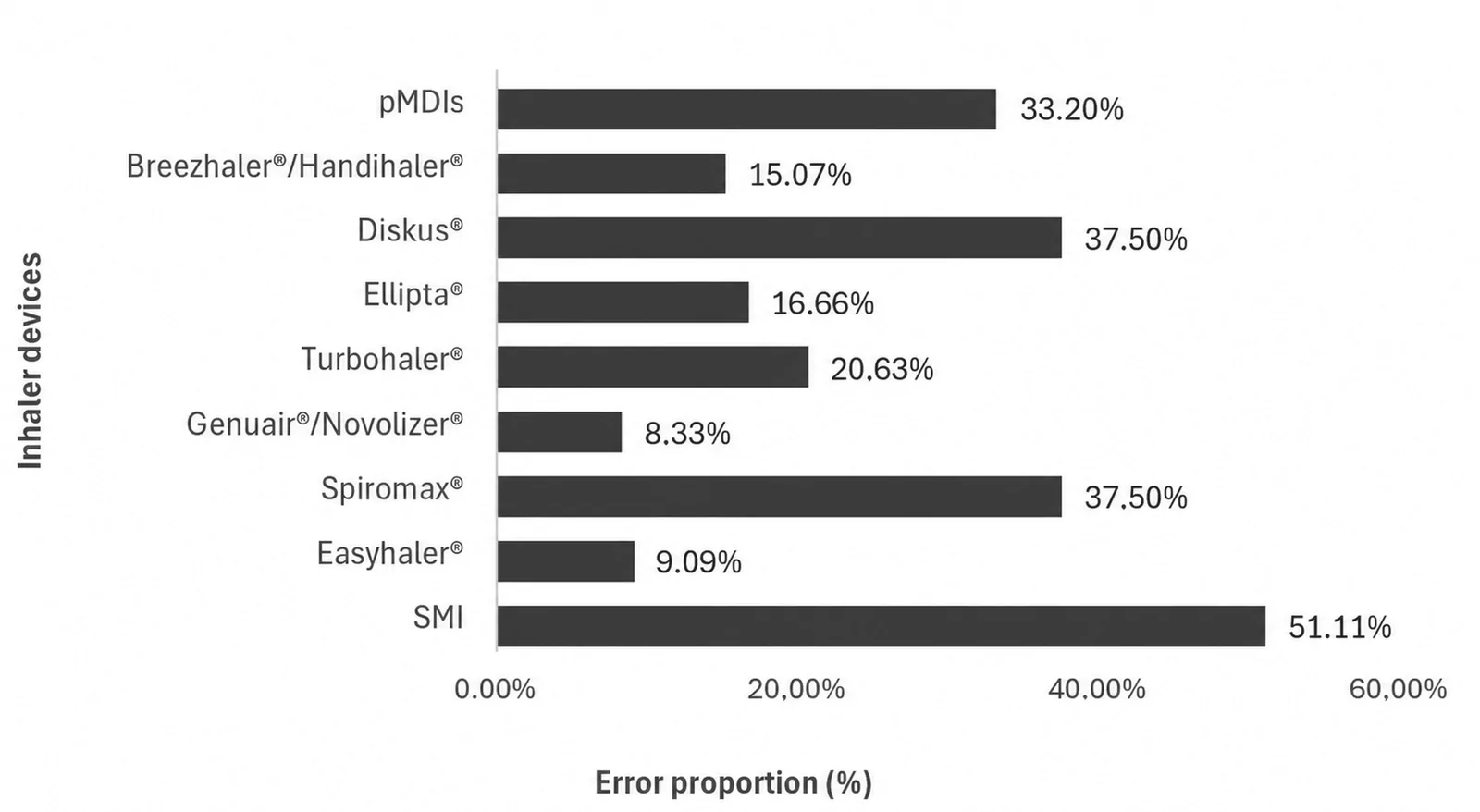
Pre-intervention error proportion. pMDIs: pressurized metered dose inhalers, SMI: soft mist inhaler.

**Figure 6:**
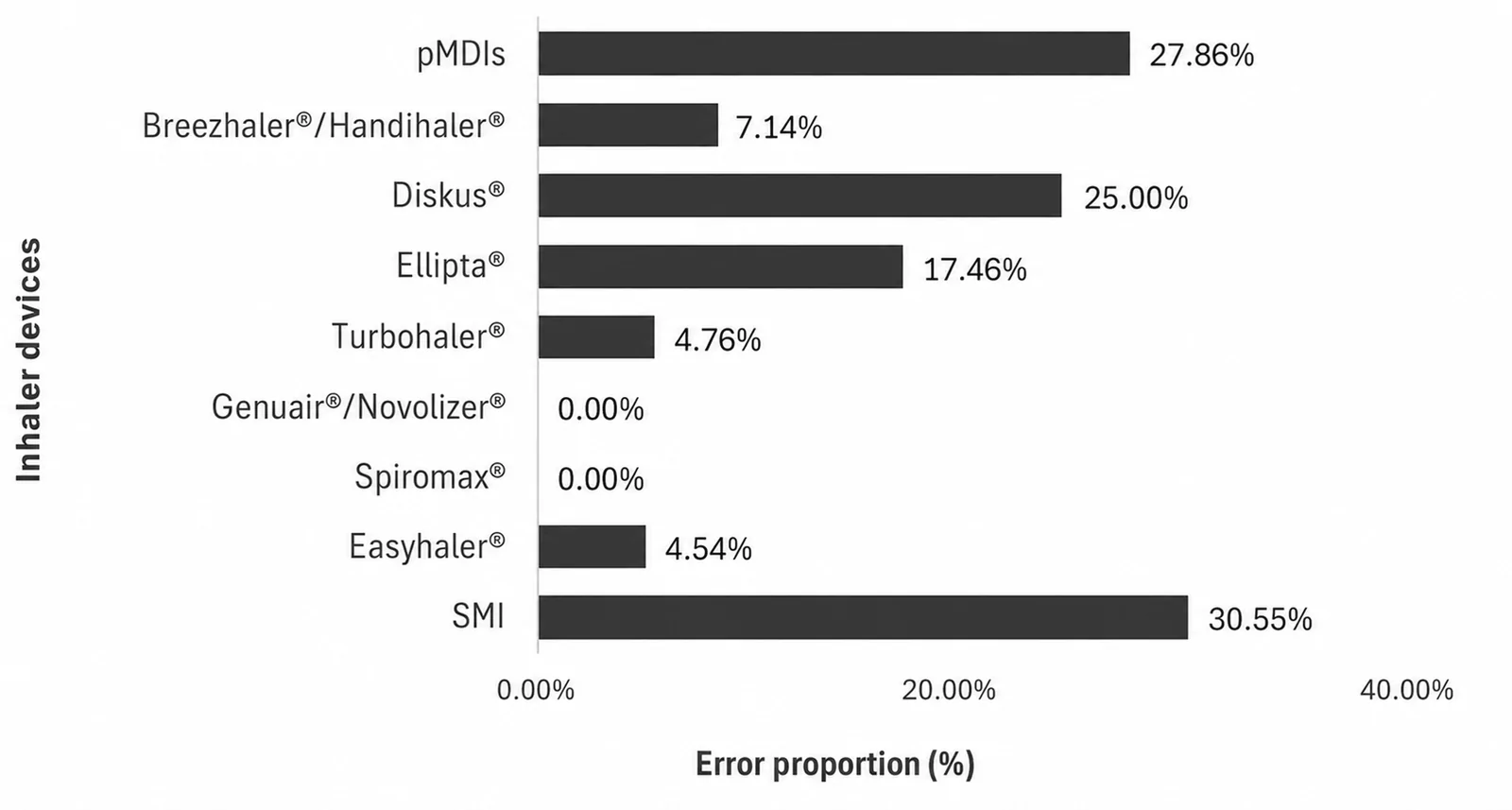
Post-intervention error proportion. pMDIs: pressurized metered dose inhalers; SMI: soft mist inhaler.

The comparison between the number of errors registered before and after the intervention, according to the inhaler device, revealed a statistically significant difference for mDPIs (p<0.001, Wilcoxon signed-rank test). Conversely, no significant improvement in error rates was observed for pMDIs, sDPIs, and SMIs (p=0.596 for pMDIs, p=0.080 for sDPIs, p=0.109 for SMIs, Wilcoxon signed-rank test).

Assessment of therapy adherence

Before the intervention, the COPD group achieved a median total score of MAUQ of 70 (IQR: 17), whereas the asthma group scored 73 (IQR: 11) out of a maximum of 112 points. Both groups exhibited the same median score in LD dimension. During the post-intervention phase, the median total score for patients with COPD was 65.5 (IQR: 11), while patients with asthma reached a median total score of 70 (IQR: 9). Figures 7, 8 illustrate the median scores obtained across each MAUQ dimension before and after the intervention for both COPD and asthma patients. The adherence assessment of individuals who underwent inhaler device changes was performed alongside their respective group.

**Figure 7:**
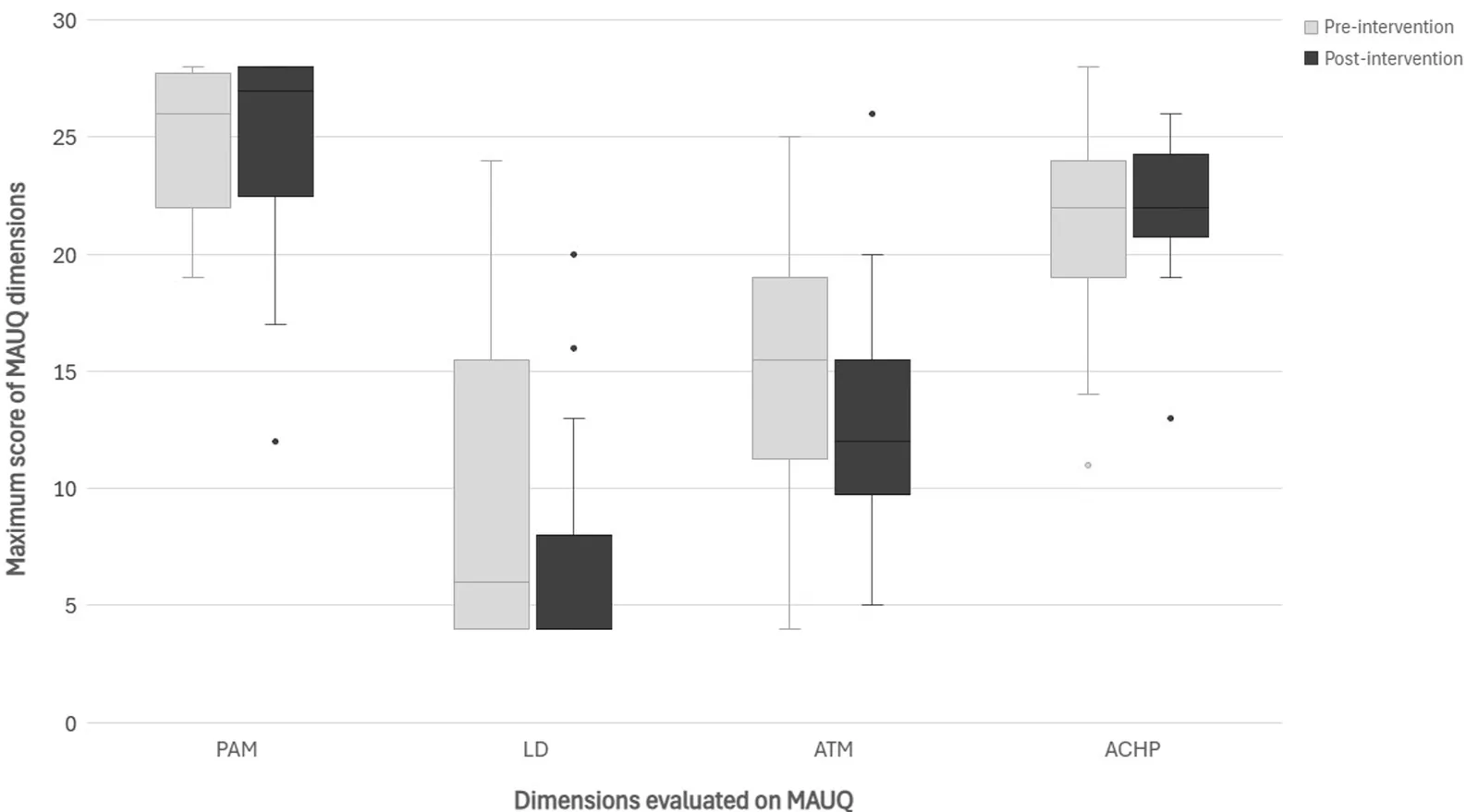
Boxplot of pre- and post-intervention MAUQ dimensions in patients with COPD. PAM: positive attitudes towards healthcare and medication; LD: lack of discipline; ATM: aversion towards medication; ACHP: active coping with health problems; MAUQ: Medication Adherence Universal Questionnaire; COPD: chronic obstructive pulmonary disease.

**Figure 8:**
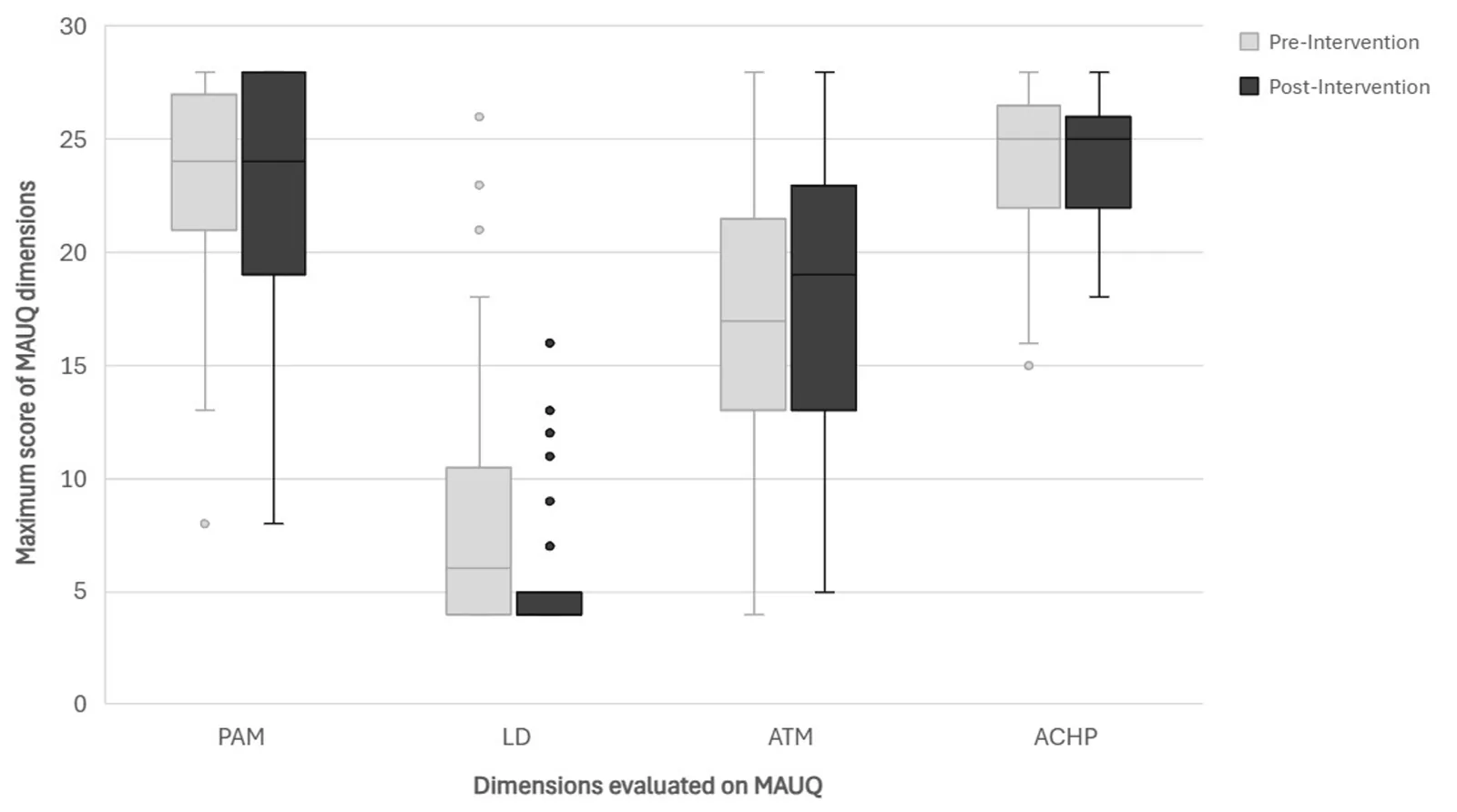
Boxplot of pre- and post-intervention MAUQ dimensions in patients with asthma. PAM: positive attitudes towards healthcare and medication; LD: lack of discipline; ATM: aversion towards medication; ACHP: active coping with health problems; MAUQ: Medication Adherence Universal Questionnaire.

The comparison of baseline and post-intervention MAUQ total scores revealed no statistically significant differences ​​​​within either group (p=0.125 for COPD, p=0.195 for asthma, Wilcoxon signed-rank test). Regarding individual MAUQ dimensions, a statistically significant improvement was noted in LD dimension before and after the intervention for both groups (p=0.041 for COPD, p=0.006 asthma, Wilcoxon signed-rank test).

## Discussion

This study highlights the clinical importance of regularly reviewing and teaching proper inhalation technique to patients with chronic respiratory diseases, such as COPD and asthma. A comparison of pre- and post-intervention common errors demonstrated significant improvements in the asthma group, particularly regarding forceful exhalation prior to actuation and holding breath for at least five seconds after inhalation. Similarly, within the COPD group, errors related to breath-holding were significantly reduced.

Regarding device-specific maneuvers, errors with pMDIs were predominantly reported among asthmatic patients, driven by the widespread use of this device type within this group. At the follow-up assessment in these patients, in five of eight (62.5%) inhalation techniques where agitation was needed, the inhaler was not shaken before use. The high rate of preparation errors in this study mirrors findings reported by Ocakli et al., who revealed that 57.30% of patients with asthma used their inhaler without shaking it beforehand. Proper agitation is crucial, as it ensures adequate homogenization of the inhalation suspension [17].

The use of pMDIs coupled with a VHC requires a different inhalation technique. In the COPD group, device-specific errors identified at baseline persisted into the post-intervention phase. These ongoing challenges align with a previous study involving patients using a pMDI-VHC combination, which reported that 80.00% did not shake the inhaler before use and 20.00% failed to connect the pMDI to the VHC [18]. The persistence of pMDI-VHC errors following face-to-face instruction highlights the clinical complexity of managing multicomponent inhalation systems in older populations. In our study, COPD patients had a median age of 70 years, a group frequently impacted by age-related dexterity challenges and reduced manual strength. The assembly of a pMDI-VHC system requires both physical dexterity and precise mechanical alignment, which directly impacts the overall inhalation technique. Consequently, clinical interventions in older COPD populations may require tailored educational strategies or simplification to less mechanically demanding devices when feasible.

Among the 12 sDPI inhalation techniques initially evaluated in the COPD group, specific errors related to capsule piercing were observed. These errors resulted from non-compliance with the checklist sequence of two consecutive steps: piercing the capsule and releasing the piercing buttons. These preparation steps were performed immediately after placing the mouthpiece in the mouth, an incorrect sequence that significantly increases the likelihood of omitting capsule piercing, executing it incorrectly, or compromising the maneuver through premature exhalation into the device, which promotes powder aggregation. Conversely, within the asthma group, the observed errors were related to loading the capsule into the device. Ideally, capsules should be loaded immediately prior to use to avoid compromising powder integrity through exposure to ambient humidity.

The Turbohaler® was the most frequently used mDPI, prescribed predominantly among asthmatic patients. At baseline, these patients frequently failed to position the inhaler upright during dose loading. The incorrect orientation, even when considering the 45-degree tolerance allowed for this device, impacts the amount of drug delivered due to the gravitational effect on the dose when leaving the reservoir [19]. Following the educational intervention, performance improved across several preparation steps. For instance, the error "Failure to twist the rotating base in one direction as far as it will go and then back in the opposite direction until a click is heard" was successfully eliminated in three evaluated techniques.

Errors with SMIs were predominantly identified during the three sequential steps of the inhalation technique associated with dose preparation. Following the educational training, these steps were performed correctly in one of the techniques within the COPD group. This baseline difficulty aligns with a systematic review by Navaie et al., which found that one in five COPD patients failed to hold the inhaler upright or were unable to rotate the base in the direction indicated by the arrows until a click was heard [20]. Conversely, within the asthma group, sequential errors regarding these initial stages persisted in one technique at the post-intervention assessment.

SMIs were associated with the highest proportion of errors at baseline, showing no statistically significant differences between the pre- and post-intervention assessments. Nevertheless, a statistically significant reduction in error rates was observed for mDPIs following the intervention. This improvement is likely attributable to their high frequency of mDPI use among patients with asthma, as these devices accounted for more than half of the devices in this group, thereby reflecting the overall improvement observed in their inhalation techniques.

The appropriate use of inhaler devices serves as a predictor of patient adherence levels. Statistical analysis revealed no significant variations in the MAUQ total scores for either group following the educational intervention. However, a dimension-by-dimension analysis demonstrated a statistically significant difference regarding the "lack of discipline" domain for both COPD and asthma patients. The LD dimension aimed to determine whether the treatment was followed consistently [16]. Baseline results for COPD suggest that forgetfulness is common among certain individuals, potentially driven by the presence of other chronic diseases that lead to polypharmacy and complex therapeutic regimens. Indeed, previous literature has reported that forgetfulness, comorbidities, and polypharmacy are significantly associated with poor adherence in individuals with COPD [12]. Following the intervention, a significant improvement in the median score of this dimension was observed, reflecting a higher level of discipline in adhering to the prescribed treatment. Regarding the asthma group, initial LD results indicated that half of the patients demonstrated consistent adherence. The post-intervention median score for this dimension also improved significantly, mirroring the results found in the COPD group. Ultimately, a high frequency of administration and complex therapeutic regimens remain major contributing factors to a lack of discipline in treatment adherence [21].

Limitations

This study has several limitations, including the small sample size, the absence of a control group, the limited statistical power, and the independence between different inhalation techniques performed by the same patient.

First, the single-center design and invitation method introduced determined the sample size. Patients were invited to participate in the study via telephone, which allowed them to decline the invitation, resulting in a high recruitment refusal rate of 68%. Recruiting participants from additional primary care units could have increased the sample size.

Second, 23 patients were lost to follow-up at two months due to scheduling conflicts, yielding an attrition rate of 30.3%. This loss to follow-up introduces potential attrition bias, as participants who completed both sessions may represent a more compliant subgroup, potentially overestimating the intervention's sustained effectiveness. The small sample size reduced statistical power, limiting the detection of subtle differences across subgroups and complicating data interpretation.

Third, the absence of a control group limited the ability to determine whether the outcomes were solely due to the educational intervention or if potential confounding factors have been involved. In order to establish true causality, future research should adopt study designs that evaluate outcomes in patients receiving standard routine care alongside patients exposed to a structured educational program led by a pharmacist or a general practitioner. Future multicenter studies incorporating control groups and longer follow-up periods are needed to establish causality and assess long-term retention. 

Finally, analyzing inhalation techniques at the device level introduces potential non-independence of results, given that individual patients could contribute multiple inhalers to the overall analysis. Patients' characteristics, such as dexterity, may have influenced technique evaluation across different devices, particularly if errors occurred in common steps and could be replicated across devices or occurred in device-specific steps for patients using more than one device within the same category.

## Conclusions

In this study, the majority of patients demonstrated handling errors when using their prescribed inhaler devices at baseline. The findings suggest that the educational intervention was associated with improved inhalation techniques, reaching statistical significance particularly among patients with asthma. Following the intervention, handling errors were significantly reduced specifically in mDPIs. In terms of therapy adherence, an improvement in the "Lack of Discipline" dimension was also observed. However, the total MAUQ score did not improve significantly, highlighting that results reflecting adherence in general must be interpreted with caution. Furthermore, given the single-arm design, single-center setting, and small sample size, direct causality cannot be firmly established, nor can these findings be generalized to broader populations.

These findings emphasize the importance of a structured educational intervention in chronic respiratory diseases within primary healthcare settings. Actionable approaches include integrating standardized inhalation assessment checklists into primary care follow-up consultations and designing peer-led educational programs to continuously upskill healthcare teams in device handling. Ultimately, combining these targeted educational measures with effective strategies to promote therapy adherence is essential to optimize patient outcomes.
